# The Insurance Bill

**Published:** 1911-05-13

**Authors:** 


### The Hospital

A Journal of

The Medical Sciences and Hospital Administration.

New Series. No. 223, Vol IX. [No. 1295, Vol. L.] SATURDAY, MAY 13 1911

### The Insurance Bill.

Both from the personal or selfish standpoint, and from that of professional experts on the question of sickness and invalidity, the Government scheme of State insurance introduced by the Chancellor of the Exchequer last week needs the close consideration of all hospital workers, and of the whole medical profession. It is true there is nothing strikingly original in the idea; as it stands it is simply a modification of the German system, which is admittedly open to improvement. That some improvements will be made during the course of the Bill through Parliament we see little reason to doubt. The disadvantage, for instance, of dealing with such widely diverse subjects as sickness and unemployment in one and the same Bill will surely become evident; and it is to be hoped that those in authority may consent to separate the two divisions into two separate Bills.

As a measure of social reform the Insurance Bill is very distinctly a step forward. The excellent effects which the German system has had upon the well-being of the community in the German Empire has often been referred to in these pages, while we have also lately dealt with the influence which it has exercised upon the German hospital system. This latter aspect of invalidity insurance is especially worth study at the present time. Our entire hospital system needs remodelling, and with it our medical service system. In this process the profession must make its voice heard, or scant attention will be paid to its rights and its grievances. The principle of co-operation between the State and the individual is recognised in the Government scheme; and it is this very principle applied to our hospital system which we have strenuously advocated as the solution of present-day problems. In so far as the Bill is an attempt to tackle one side of these problems, and notwithstanding that there are many points in it which are open to grave objection, its introduction must be hailed as a sincere effort to deal with national questions in a non-party spirit.

As anyone can easily understand from the summary of the Bill which is published in another column, there are clauses in the scheme as drafted which will radically change the aspect of private practice in certain districts. It will be seen that the existing Friendly Societies are to be conciliated by being made important factors in the working of the Act; and it is clear enough that their power and their scope are likely to be widely extended. To put the matter plainly, the medical profession has hitherto found the Friendly Societies to be, at the least, hard taskmasters, and the more affluent they get the more they tend to sweat their doctors; so that the prospect of being converted into vassals of these corporations and being deprived of all other means of existence is scarcely one which practitioners in working-class districts can face with equanimity. The Chancellor of the Exchequer expressly disclaimed the intention of imposing on the medical profession, but then he is adept at the art of conciliating in the House those who object most strongly to some of his aims and principles. The closest scrutiny will be necessary if the great societies (and none with a membership of less than 10,000 get any encouragement out of this Bill) are to be induced to treat their medical officers fairly. With the mechanism for carrying on the medical work as regards those of the insured who do *not* elect to join a Friendly Society there should be less difficulty. Here the constitution of the County Committees offers apparently some chance that the profession will get fair treatment, though even then it must bestir itself in earnest to secure it. One of the most interesting points is the provision of an allowance of thirty shillings to lying-in women; this is expressly granted on the condition that they undertake no work outside their own homes during the first four weeks of the puerperium—a most excellent proviso, but one which it will be found quite impossible to enforce. How much of this dole will go towards the doctor's fee remains to be seen; probably none at all, since most of the recipients will be attended as part of contract practice in one or other of the forms in which this Bill provides it. Another important point as affecting the profession is in respect of part-time employment. Thus, in many country districts the Medical

Officer of Health receives a salary of less than £160, though his total income from other sources may rise above that limit. Does the Bill intend that he shall profit by the insurance offered, and that his County Council must contribute its share as his employer? House surgeons at hospitals will clearly come within the provisions of the Act compulsorily. Nurses, too, are undoubtedly to be beneficiaries under the Act; and all hospital governors and proprietary nursing homes must take note of this, as it touches them financially to quite a considerable extent. Indeed, the Bill teems with matters of the closest and most vital interest to doctors and to all who are directly or indirectly in contact with the medical profession. Already, we are glad to see, the profession is deliberating its policy with regard to the Bill, and hospital workers also must not lag behind in suggesting improvements and modifications. Yet withal attention to professional interests must not blind us to the fact that the national, the communal interests are paramount in the scheme, and that it is propounded not for the benefit of medical men or hospitals, but for that of the population at large. It is necessary to lay stress on this point, for there is manifested in some quarters a tendency to attack the measure on singularly narrow lines, a tendency probably due to misconception of its scope and aims. Nothing will thus be gained. What is wanted is an impartial consideration of all sides and aspects of the scheme. With this achieved, it will be the fault of our Parliamentary representatives if injustice is done to the profession, and we look forward to results which shall give us no cause for regret, but rather for congratulation.

